# Cross‐Sectional Survey of Aflatoxin B1 Contamination in Poultry Feeds and Feed Ingredients From Bogor and Bekasi Regencies, West Java, Indonesia

**DOI:** 10.1155/vmi/1687482

**Published:** 2026-10-06

**Authors:** Raphaella Widiastuti, Romsyah Maryam, Yessy Anastasia, Prima Mei Widiyanti, Rini Damayanti, Yulvian Sani, Eny Martindah, Sutiastuti Wahyuwardani

**Affiliations:** ^1^ Research Center for Veterinary Science National Research and Innovation Agency (BRIN) Jl. Raya Jakarta-Bogor Cibinong 16911 Indonesia brin.go.id

**Keywords:** aflatoxin B1, contamination, dc-ELISA, feed ingredients, Indonesia, poultry feeds

## Abstract

Aflatoxin B1 (AFB1), a potent metabolite produced by *Aspergillus flavus* and *A. parasiticus*, poses risks to poultry production and public health. This study assessed AFB1 contamination in poultry feeds and feed ingredients collected from Bogor and Bekasi Regencies, West Java, Indonesia. The feeds were categorized across growth phases, feed forms, and sampling points. A total of 86 feed samples (37 layer feeds from Bogor Regency and 49 broiler feeds from Bekasi Regency) and 63 feed ingredients (50 corn and 13 rice bran) were analyzed using an in‐house direct competitive enzyme‐linked immunosorbent assay (dc‐ELISA). The method demonstrated high specificity (AFB1: 100%, cross‐reactivity with other aflatoxins: < 4%), strong linearity (*R* ^2^ = 0.9951 for standards; 0.9615 for spiked samples), satisfactory sensitivity (LOD: 0.45 μg/kg and LOQ: 0.77 μg/kg), and good accuracy and precision (recovery: 91.90 ± 0.81%, repeatability RSD: 1.13%). Moisture content was 11.74% in layer feeds and 9.13% in broiler feeds. AFB1 levels ranged from 0.78 to 15.45 μg/kg (median 7.00 μg/kg; IQR 4.13–9.78) in broiler feeds and 1.00–11.78 μg/kg (median 3.25 μg/kg; IQR 2.17–4.56) in layer feeds, with most values below 10 μg/kg. AFB1 contamination was nearly universal in poultry feeds (96.51%). In feed ingredients, AFB1 was detected in 78.00% of corn and 76.92% of rice bran samples, with prevalence ranging from 50.00% to 100.00% between years and commodities. The median AFB1 concentration in corn increased markedly to 126.09 μg/kg in 2024 compared to 2023. No statistically significant differences were observed across growth phases, feed forms, or sampling points [*p* > 0.05]. These findings highlight the importance of routine monitoring and targeted control of feed ingredients to sustain feed safety and protect public health in tropical regions.

## 1. Introduction

In recent years, Indonesia’s poultry industry has grown at a remarkable pace. Broiler production has now exceeded 3 billion birds, and the layer population has reached 410 million [1]. With this rapid expansion, feed safety has become a pressing issue. Aflatoxin, particularly aflatoxin B1 (AFB1), is of special concern because it directly affects animal performance including reduced hatchability and food safety due to residue occurrence in animal products [2, 3]^.^

Experimental evidence demonstrates that AFB1 induces oxidative stress, weakens intestinal integrity, and suppresses immunity, leading to reduced body weight and higher feed conversion ratios in broilers [4, 5]. High AFB1 contamination in feed (101–150 μg/kg) was observed, which impaired growth performance and raised concerns about potential residues in poultry tissues, particularly liver and muscle, by day 21 [6]. In Kalimantan, AFB1 and its metabolite AFM1 were detected in duck eggs and tissues, confirming feed‐to‐food transfer [7].

AFB1 contamination represents a serious threat to both poultry productivity and public health and therefore is classified as a Group 1 carcinogen by the International Agency for Research on Cancer (IARC) [8]. From a regulatory perspective, aflatoxins are considered unavoidable contaminants in food and feed, as current good agricultural practices cannot fully prevent or eliminate their occurrence. The maximum limit of AFB1 set by the US Food and Drug Administration (FDA) is 20 μg/kg, whereas the Indonesian government has set it at 50 μg/kg [9].

To mitigate these risks, regulatory frameworks emphasize the need for rapid, sensitive, precise, user‐friendly, and cost‐effective analytical methods for detecting aflatoxins in animal feed. Several methods, including HPLC and enzyme‐linked immunosorbent assay (ELISA), have been available for many years to identify and measure these toxins in feed and animal products [10]. ELISA remains the most widely adopted method for routine screening of aflatoxins in animal feeds due to its practicality and efficiency [11] compared to HPLC, which is used as a confirmatory method but is more resource‐intensive and less suited to routine surveillance.

In Indonesia, with tropical climates, it is highly conducive to AFB1 contamination. However, studies on AFB1 contamination and recent data on its occurrence in poultry feeds remain limited. Existing studies are often geographically restricted, focus on specific feed types or production stages, or were conducted in earlier periods, limiting their relevance to current field conditions [7, 12–15]. This highlights the need for updated and regionally representative data on AFB1 contamination in poultry feeds.

To address this gap, this study was conducted in Bogor and Bekasi Regencies, two major poultry production hubs in West Java with contrasting environmental and production characteristics. Unlike previous studies in Indonesia that were limited in scope, this study comprehensively aimed to assess AFB1 contamination in poultry feeds of both finished broiler and layer feeds across growth phases, feed forms, and different sampling points, as well as feed ingredients including corn and rice bran. The scope of this study was limited to 86 feed samples and 63 ingredient samples analyzed for AFB1 using dc‐ELISA. This approach captures multiple points of AFB1 contamination in the production chain and strengthens the basis for targeted feed safety monitoring and risk management in Indonesia.

## 2. Materials and Methods

### 2.1. Chemicals

All chemicals used in this study were analytical‐grade reagents; the solvents, including methanol, were primarily sourced from Merck (Darmstadt, Germany). The AFB1 standard was purchased from Sigma‐Aldrich (Merck KGaA, Darmstadt, Germany), and a stock solution of 1000 μg/mL was prepared in methanol. An intermediate working solution (1 μg/mL) was obtained by diluting the stock. Six calibration standards were prepared in 70% methanol by diluting the 1 μg/mL AFB1 solution to final concentrations of 0.1, 0.3, 1.0, 3.0, 10.0, and 30.0 ng/mL and then stored at −20°C.

### 2.2. Samples

In November 2023 (wet season), 25 corn and 7 rice bran samples were collected in Bogor Regency. Subsequently, in August 2024 (dry season), 86 poultry feed samples were obtained, comprising 37 layer feeds from Bogor Regency and 49 broiler feeds from Bekasi Regency, West Java, Indonesia (Figure 1), along with 25 corn and 6 rice bran samples from Bekasi. This 2‐year sampling design broadened observational coverage across varying field conditions; however, the study was not intended for formal temporal comparison, and data are therefore presented descriptively by year.

**FIGURE 1:**
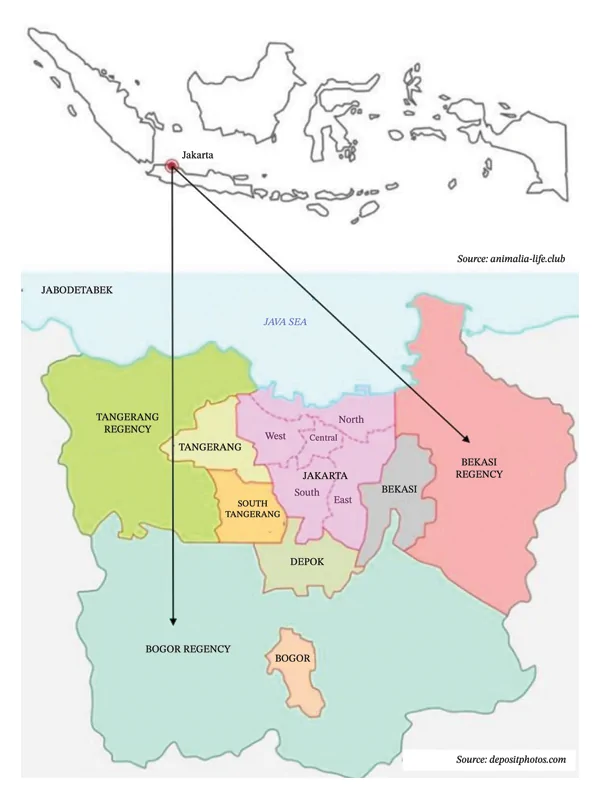
Sampling locations of Bogor and Bekasi Regencies.

At each site, samples were collected purposively and systematically during visits to minimize bias. To improve representativeness, we included diverse sources such as farm storage and retail shops. As this was an exploratory cross‐sectional study, sample size was not based on formal calculations. The target was to cover different feed types, production stages, and locations, while considering field access and laboratory capacity which guided the final sample size. Rather than being based on formal sample‐size calculations, the number of samples was determined to ensure adequate representation of commonly used poultry feeds and feed ingredients in the study areas. Practical considerations, including field accessibility and laboratory analytical capacity, also influenced the final number of samples collected. This surveillance‐oriented approach emphasizes coverage and detection of mycotoxins, rather than formal population estimates.

About 500 g samples were sealed in sterile plastic bags and transported to the National Research and Innovation Agency (NRIA) laboratory in Indonesia for analysis. To maintain sample integrity, all samples were immediately stored in a coolbox at 4°C and subsequently frozen at −20°C until laboratory analysis. Upon arrival at the laboratory, samples were promptly retrieved from frozen storage and processed to minimize storage effects. AFB1 remains relatively stable under low‐temperature conditions.

Each sample was categorized according to three predefined criteria. Growth stage referred to the production phase of the birds for which the feed was intended: starter (0–3 weeks), grower (4–6 weeks), and finisher (≥ 7 weeks). Physical feed form was classified as mash (unprocessed ground ingredients), crumble (partially compacted particles), and pellet (fully compacted in cylindrical forms). The sampling location point was defined as (i) cage leftovers—feed leftover collected directly from bird cages after feeding; (ii) farm warehouse—bulk feed stored in farm‐level storage facilities prior to use; and (iii) retail poultry shop—commercially packaged feed purchased from local vendors/manufacturers. This classification allowed us to assess contamination risks across different handling conditions and production phases.

### 2.3. Sample Extraction and Analysis by ELISA

#### 2.3.1. Sample Extraction

All feed samples were ground, homogenized, and subsampled before AFB1 extraction. Twenty‐five‐gram samples were extracted with 100 mL of 70% methanol on a shaker (Gallenkamp, Germany) for 30 min. The extract was filtered through Whatman #41 filter paper.

#### 2.3.2. Validation of ELISA Method

The ELISA kit was prepared in the laboratory, comprising 96‐well microplates (both antibody‐coated and uncoated), six concentrations (0.1, 0.3, 1.0, 3.0, 10.0, and 30.0 ng/mL) of AFB1 standards in 70% methanol, an enzyme conjugate solution, substrate solutions, and a stop solution. The method was fully validated by evaluating its performance for specificity, linearity, sensitivity, accuracy, and precision prior to sample analysis [16]. Performance characteristics which are presented in Table 1 were evaluated with appropriate measures of variability prior to sample analysis.

**TABLE 1:** Performance characteristics of the ELISA method for AFB1 detection in this study.

Performance characteristics	Result of measurement	Evaluation	Reference	Remark
Specificity	Cross‐reactivity: AFB1 = 100.00% AFB2 = 1.95% AFG1 = 3.23% AFG2 = 2.13%	Cross reactivity against other analytes < 4%	[18]	Accepted
Linearity	*R* ^2^ = 0.9951 (AFB1 standard) *R* ^2^ = 0.9618 (in sample matrix)	*R* ^2^ ≥ 0.85	[18]	Accepted
Sensitivity	LOD = 0.45 ng/kg LOQ = 0.77 ng/kg	< 5% nominal value	[18]	Accepted
Accuracy	Recovery = 91.90%	Recovery: 70%–125%	[17]	Accepted
Precision	Repeatability: • RSD = 0.013 • CV = 1.13 (CV_Hor_ = 1.97)	CV < 2/3 CV Horwitz RSD < 30%	[17]	Accepted

Specificity performance of the method was assessed by cross‐reactivity with AFB1 and other aflatoxins, such as AFB2, AFG1, and AFG2. The cross‐reactivity of each toxin was calculated using the equation Rx = (IC50 AF/IC50 AFB1) × 100% and evaluated according to the standard guideline [17].

Linearity of the method was measured using six concentrations of the AFB1 standard (0.1, 0.3, 1.0, 3.0, 10.0, and 30.0 ng/mL) as the working solution and by analyzing spiked feed samples with the AFB1 standard at the same concentrations. The working solutions were prepared by diluting the AFB1 stock to 1 μg/mL (1 ppm). Prior to analysis, all samples were subjected to a standard fourfold dilution. For samples exhibiting elevated analyte concentrations, additional dilution steps were performed to ensure values remained within the validated detection range of the assay. Linearity was evaluated by comparing the R2 values of the calibration curve with the standard guideline [18].

The limit of detection (LOD) was determined by analyzing AFB1 in spiked feed samples at the lowest concentration (*n* = 10) and was calculated as the mean signal plus 3 SD, while the limit of quantitation (LOQ) was set at the mean signal of the blanks plus 10 SD. Values below the LOD were reported as not detected (ND).

The precision of the method was evaluated by analyzing a naturally contaminated feed sample (*n* = 10), calculating the RSD, and comparing it with the AOAC guidelines [17]. The accuracy of the method was evaluated by spiking blank feed matrices with AFB1 standard at three concentration levels (low, medium, and high) within the linear range, each analyzed in six replicates (*n* = 6). The recovery percentage was calculated by comparing the measured concentration to the spiked concentration. Accuracy was considered acceptable if the recovery values were within the range of 70%–120% according to AOAC guidelines.

The dc‐ELISA kit showed high specificity for AFB1 (100%) with minimal cross‐reactivity (AFB2 1.95%, AFG1 3.23%, and AFG2 2.13%). Linearity of the method was indicated by the coefficient of correlation for the AFB1, which was strong (*R* ^2^ = 0.9951 for standards; 0.9615 for spiked samples). Sensitivity was indicated by LOD 0.45 μg/kg and LOQ 0.77 μg/kg. The accuracy was 91.9 ± 0.81%, while the precision was reflected by RSD 1.13%.

#### 2.3.3. Sample Analysis by ELISA

AFB1 concentrations in feed samples were analyzed using direct competitive ELISA. All test samples were brought to room temperature before analysis. An uncoated microplate was prepared by adding 100 μL of conjugate to each well, followed by 100 μL of either standard solution or sample extracts, mixing with a multichannel micropipette, and then transferring 100 μL (for single test) or 75 μL (for duplicate tests) to the antibody‐coated wells. The plate was incubated for 30 min, washed three times with distilled water, and air‐dried. Subsequently, 100 μL of the substrate solution was added to each well, incubated for 15 min, and then 50 μL of stop solution was added. The absorbance was measured at 450 nm using an ELISA reader, and AFB1 concentrations were calculated based on the standard curve equation.

### 2.4. Statistical Analysis

ANOVA was not applied in the final analysis. Instead, nonparametric tests (Kruskal–Wallis) were used for group comparisons, as they are more appropriate for skewed data. The Mann–Whitney *U* test was used to compare AFB1 concentrations between two independent groups (layer *vs* broiler feeds), while the Kruskal–Wallis test was used for comparisons across more than two groups (e.g., growth stage, feed form, and storage location). A *p* value < 0.05 was considered statistically significant.

Potential confounding variables, including feed type, sampling year, and sampling location, were considered during data interpretation. Due to the exploratory nature of the study and the distribution of the sample across categories, no multivariable statistical modeling was performed. Instead, results are presented using stratified analyses to allow comparison across relevant categories. The ND values were excluded from statistical analyses.

Variability was expressed as IQR for skewed data. All data in this study showed a nonnormal distribution; therefore, median, and IQR were reported throughout. Confidence intervals were not reported because this study employed a convenience sampling approach from specific regencies and poultry shops and was not designed to infer results to a broader population. Therefore, descriptive statistics are presented to summarize the data distribution within the sampled population. Analyses were conducted at a 95% confidence level (*α* = 0.05) using SPSS Version 20 (IBM Corp., Armonk, NY).

## 3. Results

### 3.1. Moisture Contents and AFB1 Contamination in Poultry Feed Samples

Moisture content of a feedstuff is a good marker of the quality and shelf life of grains. During sample collection, the ambient temperatures in Bogor Regency ranged from 30°C to 32°C, and feed sample moisture levels ranged from 4.46% to 22.00%, averaging 11.74%. In contrast, Bekasi Regency experienced temperatures ranging from 33°C to 35°C and moisture content ranging from 7.33% to 11.17%, averaging 9.13%.

The occurrence, moisture content, and AFB1 concentration in all poultry feed samples from Bogor and Bekasi Regencies are summarized in Table 2 and Figure 2. The median AFB1 contamination level of all samples was 4.78 μg/kg (IQR: 3.25–7.54). These differences are relevant because elevated moisture, particularly above 12%–13%, combined with warm tropical conditions, creates a conducive environment for *Aspergillus flavus* proliferation and subsequent AFB1 contamination. Thus, documenting moisture levels provides critical context for interpreting contamination risks across regions.

**TABLE 2:** Occurrence and concentration of AFB1 in poultry feeds from Bogor and Bekasi Regencies.

Regency	Feed type	*n*	Moisture content (%)	Positive AFB1 (%)	Range (μg/kg)	Median (IQR) (μg/kg)
Bogor	Layer	37	11.74	91.90	1.00–11.78	3.34 (2.17–4.80)
Bekasi	Broiler	49	9.13	100.00	0.78–15.45	7.00 (4.05–8.76)
Total		86	10.25	96.51	0.78–15.45	4.78 (3.25–7.54)

*Note:* Values are presented as median (IQR). IQR: interquartile range (P25–P75). Superscript letters (^a^,^b^) denote significant differences between regencies for each parameter (*p* < 0.05, Mann–Whitney *U* test). The maximum permissible level of AFB1 in poultry feed in Indonesia is 50 μg/kg.

Abbreviation: ND, not detected.

**FIGURE 2:**
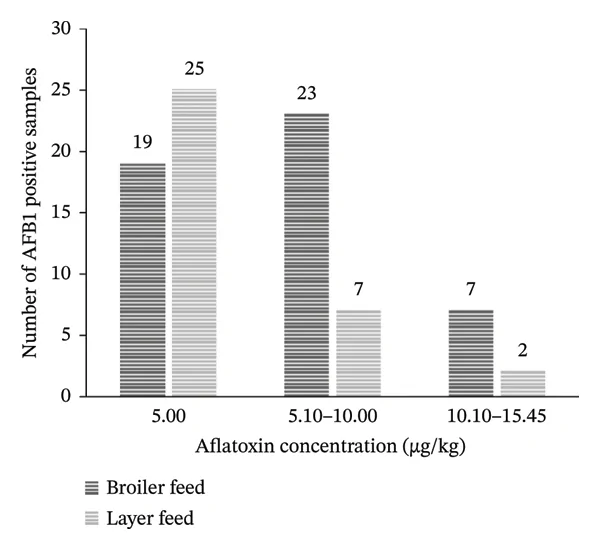
Distribution of positive AFB1 at various concentration levels in broiler and layer poultry feeds.

The most prevalent AFB1 contamination in both types of poultry feed was below 10 μg/kg. AFB1 was detected in 91.89% of layer feed samples from Bogor and in all broiler feed samples (100%) from Bekasi. The overall occurrence of AFB1 contamination across all samples was 96.51%. AFB1 concentrations in broiler feeds (median: 7.00 μg/kg, IQR: 4.13–9.78) were higher and more variable than those in layer feeds (median: 3.25 μg/kg, IQR: 2.17–4.56). Median values provide a more reliable measure of central tendency given the nonnormal distribution of the data.

A nonparametric comparison (Mann–Whitney *U* test, *p* = 0.012) confirmed a statistically significant difference between broiler and layer feeds, with median contamination levels consistently higher in broiler feed, likely reflecting their composition, as maize and protein‐rich ingredients are more prone to fungal growth and aflatoxin production [2]. The median AFB1 contamination observed in this study, 4.78 μg/kg (3.25–7.54), was lower than reported levels in Pakistan (34–86.2 μg/kg) [10], which may be attributed to inadequate storage awareness, insufficient ventilation, and poor temperature control at farms.

### 3.2. AFB1 Contamination in Poultry Feeds

#### 3.2.1. The occurrence of AFB1 in Different Growth Stages of Chicken Feeds

Feeds were classified according to poultry growth stage (starter, grower, and finisher) for detailed analysis of AFB1 contamination. Table 3 presents the occurrence and concentration levels of AFB1 contamination in chicken layer feeds across different growth stages (starter, grower, and finisher).

**TABLE 3:** Occurrence and concentration of AFB1 across growth stages of poultry feeds.

Feed type	Growth stage	*n*	AFB1 positive (%)	Range (μg/kg)	Median (IQR) (μg/kg)
Layer feed	Starter	7	100.00	0.78–10.75	3.24 (2.05–3.81)
	Grower	17	88.23	1.20–15.30	3.34 (2.17–4.56)
	Finisher	13	92.31	3.95–11.75	3.78 (2.77–6.39)

Broiler feed	Starter	9	100.00	1.99–11.78	2.42 (0.78–4.05)
	Grower	20	100.00	1.48–9.78	4.65 (3.89–8.14)
	Finisher	20	100.00	1.00–11.58	8.01 (7.18–9.78)

*Note:* Values are presented as median (IQR). IQR: interquartile range (P25–P75). The maximum permissible level of AFB1 in poultry feed in Indonesia is 50 μg/kg.

Abbreviation: ND, not detected.

AFB1 contamination across different growth stages of chicken feeds is presented in Table 3. In layer feeds, AFB1 was widely detected, with occurrence rates of 100% in starter, 88.23% in grower, and 92.31% in finisher feeds. The median concentration showed an increase from 2.42 μg/kg (IQR: 0.78–4.05) in the starter to 8.01 μg/kg (IQR: 7.18–9.78) in the finisher. In broiler feeds, AFB1 was detected in all samples across starter, grower, and finisher phases (100% occurrence). Median values indicated relatively stable central tendencies, ranging from 3.24 μg/kg (IQR: 2.05–3.81) in starter to 3.78 μg/kg (IQR: 2.77–6.39) in finisher.

Due to the nonnormal distribution of the data, AFB1 concentrations across growth stages were analyzed using the Kruskal–Wallis test. The test showed no statistically significant difference in median AFB1 concentration among the three growth stages (*p* = 0.095). Nonetheless, the consistently high prevalence in both layer and broiler feeds indicates that growth stage does not mitigate AFB1 risk, warranting continuous monitoring.

#### 3.2.2. The occurrence of AFB1 in Different Forms of Finished Feeds

The physical form of poultry feed is key in optimizing bird growth performance and body weight, which are essential for both market value and consumption. Chicken feed commonly comes in mash, pellet, or crumble forms. The selection of the type of finished feed to use is influenced by the farmer’s production goals and the current economic conditions [19]. AFB1 contamination across different feed forms is presented in Table 4.

**TABLE 4:** Occurrence and concentration of AFB1 in poultry feeds across different feed forms.

Feed forms	Feed forms	*n*	AFB1 positive (%)	Range (μg/kg)	Median (IQR) (μg/kg)
Layer feed	Mash	17	100.00	1.48–11.78	3.30 (2.27–4.38)
	Crumble	15	80.00	1.00–11.58	4.44 (2.70–6.45)
	Pellet	5	100.00	3.00–5.85	3.99 (3.22–5.85)

Broiler	Mash	4	100.00	0.78–9.58	4.03 (2.96–5.41)
	Crumble	37	100.00	1.20–15.45	7.43 (4.13–9.21)
	Pellet	8	100.00	1.20–11.64	6.47 (5.85–8.29)

*Note:* Values are presented as median (IQR). IQR: interquartile range (P25–P75). The maximum permissible level of AFB1 in poultry feed in Indonesia is 50 μg/kg.

Abbreviation: ND, not detected.

In layer feeds, median values of AFB1 showed that crumble feed had the highest central tendency (median: 4.44 μg/kg; IQR: 2.70–6.45), followed by pellet (3.99 μg/kg; IQR: 3.22–5.85) and mash feeds (3.30 μg/kg; IQR: 2.27–4.38). In broiler feeds, AFB1 contamination was also widely detected, although occurrence was slightly lower in crumble feeds (80%) than in mash and pellet feeds (100%). Median concentrations were highest in crumble feeds (7.43 μg/kg; IQR: 4.13–9.21), followed by pellet (6.47 μg/kg; IQR: 5.85–8.29) and mash feeds (4.03 μg/kg; IQR: 2.96–5.41).

Due to nonnormal distribution of the data, AFB1 concentrations among different feed forms were analyzed using the Kruskal–Wallis test. No statistically significant differences were observed among feed forms (*p* = 0.320). Therefore, the observed tendencies should be interpreted as descriptive patterns rather than confirmed effects.

#### 3.2.3. AFB1 in Samples at Different Sampling Points

Storage conditions, such as warm temperature and humid environments, greatly influence the differences in aflatoxin content in feed [2]. Indonesia’s tropical climate, characterized by high humidity, created an ideal environment for the growth of *A. flavus*, which triggers the formation of AFB1. Smallholder farmers contributed to the rising aflatoxin contamination of feed, primarily due to limited understanding and awareness of aflatoxin, leading to suboptimal livestock management practices [20]. AFB1 contamination in poultry feeds varied across sampling points as shown in Table 5.

**TABLE 5:** Occurrence and concentration of AFB1 in poultry feeds at different sampling points.

Feed type	Feed categories	*n*	AFB1 positive (%)	Range (μg/kg)	Median (IQR) (μg/kg)
Layer feed	Leftover in the cage	9	100.00	1.48–6.25	3.67 (1.99–3.99)
	Stored in warehouse	7	100.00	1.91–6.93	3.25 (2.54–4.80)
	Sold in a poultry shop	21	85.71	1.0–11.78	3.53 (2.15–5.85)

Broiler feed	Leftover in the cage	21	100.00	2.59–15.30	7.40 (4.28–8.76)
	Stored in warehouse	17	100.00	2.55–15.45	6.53 (4.45–9.21)
	Sold in a poultry shop	11	100.00	0.78–12.11	3.37 (1.20–9.82)

*Note:* Values are presented as median (IQR). IQR: interquartile range (P25–P75). The maximum permissible level of AFB1 in poultry feed in Indonesia is 50 μg/kg.

Abbreviation: ND, not detected.

In layer feeds, AFB1 was detected in 100% of leftover feed in the cage and warehouse‐stored feed and in 85.71% of feed sold in poultry shops. Median concentration of AFB1 was comparable, ranging from 3.25 (IQR: 2.54–4.80) in warehouse‐stored feed to 3.67 μg/kg (IQR: 1.99–3.99) in leftover feed. In broiler feeds, AFB1 was detected in all samples (100%) across all sampling points. The highest median concentration was observed in leftover feed in the cage (7.40 μg/kg; IQR: 4.28–8.76), followed by warehouse‐stored feed (6.53 μg/kg; IQR: 4.45–9.21) and feed sold in poultry shops (3.37 μg/kg; IQR: 1.20–9.82).

Despite lower median values, poultry shop samples showed substantial variability, suggesting heterogeneous contamination levels. Overall, AFB1 contamination was consistently present across all sampling points in both production systems, with differences primarily reflected in variability rather than clear differences in central tendency.

Because the data were not normally distributed, we used the Kruskal–Wallis test to compare AFB1 levels across sampling points. The results showed no statistically significant difference in median AFB1 concentration among sites (*p* = 0.108). This suggests that AFB1 contamination was detected consistently across all sampling points. We should therefore treat these contextual factors as potential contributors rather than confirmed causes. The findings highlight the need for careful interpretation and further study under controlled conditions.

### 3.3. AFB1 Contamination in Feed Ingredients

AFB1 contamination in poultry feed ingredients, especially corn, poses a substantial constraint on achieving efficient animal protein production [21]. Corn was identified as a major contributor to aflatoxin contamination in feeds [22]. The occurrence and concentration level of AFB1 contamination in maize and rice bran collected from poultry shops in October 2023 and August 2024 are presented in Tables 6 and 7, respectively.

**TABLE 6:** Occurrence and concentration of AFB1 in corn across sampling years.

Year	Number of samples	AFB1 positive, *n* (%)	Range (μg/kg)	Median (IQR) (μg/kg)
2023	25	23 (92.00)	0.19–11.42	2.24 (0.42–5.13)
2024	25	16 (64.00)	2.06–671.98	126.09 (39.48–268.73)
Total	50	39 (78.00)	0.19–671.98	8.50 (1.20–145.60)

*Note:* Values are presented as median (IQR). IQR: interquartile range (P25–P75). The maximum permissible level of AFB1 in poultry feed in Indonesia is 50 μg/kg.

Abbreviation: ND, not detected.

**TABLE 7:** Occurrence and concentration of AFB1 in rice bran across sampling years.

Year	Number of samples	AFB1 positive, *n* (%)	Range (μg/kg)	Median (IQR) (μg/kg)
2023	7	7 (100.00)	0.33–12.82	7.27 (2.62–10.13)
2024	6	3 (50.00)	0.53–38.03	1.53 (0.53–38.03)
Total	13	10 (76.92)	0.33–38.03	5.40 (0.53–10.13)

*Note:* Values are presented as median (IQR). IQR: interquartile range (P25–P75). The maximum permissible level of AFB1 in poultry feed in Indonesia is 50 μg/kg.

Abbreviation: ND, not detected.

AFB1 contamination in feed ingredients was widespread, with prevalence ranging from 50.00% to 100.00% depending on the commodity and sampling year. In 2023 samples were collected from Bogor, while 2024 samples originated from Bekasi. Therefore, observed differences may reflect not only seasonal or environmental conditions but also regional variation in feed production and storage practices.

In corn, AFB1 was detected in 39 of 50 samples, resulting in an overall prevalence of 78.00%. The prevalence decreased from 92.00% in 2023 to 64.00% in 2024. The AFB1 concentration ranged from 0.19 to 671.98 μg/kg. The overall median concentration was 8.50 μg/kg (IQR: 1.20–145.60). The median concentration was substantially higher in 2024 [126.09 μg/kg; IQR: 39.48–268.73] compared to 2023 (2.24 μg/kg; IQR: 0.42–3.13), and several samples exceeded the maximum permissible limit of 50 μg/kg, which can cause severe poultry health issues and pose a potential risk of aflatoxin residues.

In rice bran, AFB1 was detected in 10 of 13 samples, with an overall prevalence of 76.92%. The prevalence was 100.00% in 2023 and decreased to 50.00% in 2024. The concentration in 2024 ranged from 0.53 to 38.03 μg/kg, with a median of 1.53 μg/kg (IQR: 0.53–38.03), and both below the regulatory limit. The overall median concentration was 5.40 μg/kg (IQR: 0.53–10.13) for all years combined.

## 4. Discussion

Mycotoxin contamination of poultry feed and feed ingredients were observed, posing significant problems for poultry production and human health. Elevated moisture content and AFB1 levels in stored feed ingredients are strongly influenced by tropical climatic conditions, particularly high humidity and temperature, accounting for 60%–70% of the variation in fungal growth and aflatoxin production in tropical regions [23]. Whereas poor postharvest practices and prolonged storage contribute 20%–30% of the contamination risk, fungal strain, and substrate type [24] play a secondary but notable role.

Moisture content in Bogor (11.74%) was higher than in Bekasi (9.13%), with an overall average of 10.25%. Although these values remain below the Indonesian Standard Authority’s 14% [9], a threshold for fungal growth and AFB1 formation [25], Bogor’s proximity to this limit underscores its greater contamination risk. This highlights the importance of monitoring to reduce the risk of AFB1 contamination [26]. The moisture content of the samples was lower than that reported in Peninsular Malaysia, where values ranged from 6.5% to 27.3% across 336 poultry feed samples [27].

In this study, 96.51% of samples were positive for AFB1, with most contamination levels below 10 μg/kg. The median AFB1 contamination level was 3.53 μg/kg (IQR: 2.17–7.00), which remained below the Indonesian regulatory limit of 50 μg/kg [9]. The higher median AFB1 concentrations observed in broiler feeds suggest a greater contamination burden than in layer feeds, likely due to higher maize content and the presence of *Aspergillus flavus* under tropical storage conditions [28]. Although concentrations were generally low, chronic exposure, even at subregulatory levels, can impair poultry performance and increase residue risks in animal products, underscoring the importance of continuous monitoring for both poultry health and food safety.

AFB1 levels differed across growth stages, and broiler feeds generally had higher contamination than layer feeds. However, the interaction between feed type and growth stage was not statistically significant (*p* = 0.274). This suggests the increase in AFB1 with growth stage was similar in both groups.

Reporting median and IQR values helped show the variability within each growth stage. Differences across countries [29–31] suggest that AFB1 contamination reflects mechanistic factors such as feed composition, storage duration, climatic conditions, and the extent of regulatory enforcement. For example, higher contamination in broiler feeds may relate to maize‐rich diets and shorter storage cycles, while the consistently high levels in Pakistan may reflect a climate that supports *Aspergillus* growth. Overall, these results show that contamination patterns depend not only on feed type and stage but also on local management practices.

Crumble feeds exhibited the highest median AFB1 levels, which contrast with previous reports in pellet feeds [32, 33]. This may be influenced by the inclusion of toxin binders commonly added during pelleting, complicating interpretation. Although, pelletizing with steam combined with binders at a total level of 1.2% reduced AFB1 levels by 41% [34], binders can interfere with analytical detection by adsorbing toxins, potentially masking true contamination [35, 36]. This dual effect underscores the need to consider additive use when comparing feed forms and suggesting that physical processing may not reduce aflatoxin content and could concentrate toxins.

Prolonged feed storage (more than 21 days) [37] increases fungal growth and the risk of AFB1. The results in Table 4 showed that broiler feeds consistently showed higher contamination than layer feeds, with shop‐stored feed reaching the highest levels. In broiler feeds, higher median AFB1 concentrations in leftover and warehouse samples may be linked to prolonged exposure to environmental conditions, such as humidity and temperature, that favor fungal growth and toxin production. In layer feeds, the slightly lower occurrence observed in poultry shop samples compared to farm‐based samples may reflect differences in storage duration, handling, or turnover rates at the retail level. However, the relatively similar median concentrations across locations suggest that the storage site alone does not strongly determine contamination levels. In contrast, poultry shop feeds showed lower median values but greater variability, indicating inconsistent storage and handling during distribution.

Despite these observed patterns, the lack of statistically significant differences suggests that storage location is not the sole determinant of AFB1 contamination. Rather, contamination likely results from multiple interacting factors, including initial ingredient quality and humid tropical climates, which remain key contributors to aflatoxin contamination in Indonesia. Practically, contamination risks exist throughout the supply chain, so monitoring should cover all stages. These findings indicate that, even in the absence of statistically significant differences, storage practices remain a potential risk for aflatoxin contamination in poultry feed. Poor feed storage practices, such as placing feed directly on the floor and inadequate hygiene, are significant contributors to aflatoxin contamination [38], as observed in different locations in Indonesia due to poor storage facilities and humid tropical climates [13–15]. A study conducted in Kenya found no significant association between specific feed‐handling practices and mycotoxin levels, including storage location or the elevation of feed sacks [39]. However, proper storage practices, such as placing feed bags on dry wooden platforms and ensuring they are adequately covered, can effectively minimize contamination risks [40].

Although the study focused on poultry feeds, contamination in feed ingredients including corn and rice bran, poses a direct risk for carry‐over into finished poultry feed and subsequently into animal products. In feed ingredients, AFB1 contamination showed a distinct temporal pattern and differed between commodities.

In corn, AFB1 was detected in 78.00% of samples overall. The prevalence decreased from 92.00% in 2023 to 64.00% in 2024. However, the median concentration increased substantially in 2024 [126.09 μg/kg; IQR: 39.48–268.73] compared to 2023 [2.24 μg/kg; IQR: 0.42–3.13], with a maximum level of 671.98 μg/kg. This suggests that while fewer corn samples were contaminated in 2024, those that were contaminated had much higher AFB1 levels. Several samples exceeded the maximum permissible limit of 50 μg/kg, indicating a potential risk for poultry health and aflatoxin carry‐over, which may be attributed to poorer storage conditions or higher humidity during the 2024 sampling period.

In rice bran, AFB1 was detected in 76.92% of samples overall. The prevalence was 100.00% in 2023 and decreased to 50.00% in 2024. The median concentration in 2024 was 1.53 μg/kg (IQR: 0.53–38.03), with a maximum of 38.03 μg/kg, and all values were below the regulatory limit of 50 μg/kg. The overall median of 5.40 μg/kg suggests rice bran poses a lower risk of AFB1 contamination than corn in this study, although continuous monitoring is still needed.

Seasonality may play a role in AFB1 contamination, but regional differences are also important. Samples from Bogor in 2023 and Bekasi in 2024 represent an additional confounding variable. Different supply chains and storage practices can affect temporal comparisons. To address this, data from the 2 years were presented separately as Tables 6 and 7 to allow for clearer interpretation and made the results easier to interpret.

These differences show the importance of broader multisite surveillance before linking contamination patterns directly to environmental conditions. Overall, our data support regular monitoring of both prevalence and concentration, particularly in tropical areas. The results match a previous study from Java Island, Indonesia, which also found high AFB1 rates in wheat bran [100%], rice bran [100%], and maize [83.3%] collected from feed stores, silos, and bins [10].

Given the consistently high prevalence of AFB1 contamination in poultry feeds [96.55%] and the variable but substantial occurrence in feed ingredients [prevalence range: 50.00%–100.00%], routine monitoring and targeted control of feed ingredients are essential to sustain feed safety and protect public health in tropical regions.

These findings reinforce the need for comprehensive control strategies, including routine monitoring by veterinary authorities to detect contamination early, Hazard Analysis and Critical Control Point (HACCP) implementation in feed mills to strengthen preventive measures, and farmer education on storage and handling to directly mitigate local risk factors. Furthermore, combined with toxin binders in feed formulations, they provide an evidence‐based framework to reduce mycotoxin contamination [3] and safeguard poultry health in the regions.

## 5. Conclusions

AFB1 was found in 96.51% of poultry feed samples. Most were below 10 μg/kg and thus within the 50 μg/kg regulatory limit. Statistical analysis showed no significant differences in AFB1 across feed forms, sampling points, or growth phases. This suggests contamination levels were consistent across handling conditions.

High contamination was also seen in feed ingredients. In corn, prevalence decreased from 92.00% in 2023 to 64.00% in 2024. Rice bran showed a similar decrease, from 100.00% to 50.00%. However, corn contamination became much worse in 2024. The median AFB1 level increased from 2.24 μg/kg in 2023 to 126.09 μg/kg in 2024, with the maximum recorded value reaching 671.98 μg/kg, far exceeding the regulatory limit of 50 μg/kg.

These results indicate that risk depends not only on the frequency of contamination but also on concentration levels. To protect feed safety and public health in tropical regions, stricter control is needed. This should include better pre‐ and postharvest handling, proper storage, and regular monitoring of feed ingredients.

## 6. Limitation

This study faced constraints in obtaining a randomized, diverse, and evenly distributed sample set. These limitations may impact the extent to which the findings can be applied to other regions or types of feed. As a result, there’s a possibility of sampling bias or inconsistencies that could influence the interpretation of the contamination patterns**.**

## Author Contributions

Raphaella Widiastuti: conceptualization, investigation, writing–original draft, methodology, validation, visualization, writing–review and editing, formal analysis, data curation, and supervision; Romsyah Maryam: conceptualization, investigation, funding acquisition, writing–original draft, methodology, validation, visualization, writing–review and editing, project administration, data curation, supervision, and resources; Yessy Anastasia: investigation, writing–original draft, validation, methodology, visualization, writing–review and editing, data curation, formal analysis, and software; Prima Mei Widiyanti: validation, methodology, writing–original draft, software, formal analysis, data curation, and investigation; Rini Damayanti: writing–original draft, conceptualization, visualization, writing–review and editing, data curation, and software; Yulvian Sani: conceptualization, writing–original draft, visualization, writing–review and editing, data curation, software; Eny Martindah: conceptualization, writing–original draft, writing–review and editing, software, data curation, visualization, supervision, formal analysis; and Sutiastuti Wahyuwardani: conceptualization, writing–original draft, visualization, writing–review and editing, software, formal analysis, data curation, and supervision.

## Funding

This study was supported by the National Research and Innovation Agency, Republic of Indonesia, Health Research Organization, under “Rumah Program Purwarupa Alat Deteksi Kesehatan” of the Grants No.16/III.9/HK/2023 for the fiscal year 2023 and No.6/III.9/HK/2024 for the fiscal year 2024.

## Ethics Statement

Ethical approval was not required for this study.

## Conflicts of Interest

The authors declare no conflicts of interest.

## Data Availability

The data that support the findings of this study are available from the corresponding author upon reasonable request.
